# Altered Th17/Treg Ratio in Nasal Polyps With Distinct Cytokine Profile: Association With Patterns of Inflammation and Mucosal Remodeling

**DOI:** 10.1097/MD.0000000000002998

**Published:** 2016-03-11

**Authors:** Dawei Wu, Jianting Wang, Min Zhang

**Affiliations:** From the Department of Otorhinolaryngology, Head and Neck Surgery, Beijing Chaoyang Hospital, Capital Medical University, Beijing, China.

## Abstract

Chronic rhinosinusitis with nasal polyps (CRSwNP) is a heterogeneous disease that can be classified as eosinophilic or noneosinophilic. Nasal polyps can exhibit different types of mucosal inflammation and responses to treatment. Imbalanced ratios of T-helper 17(Th17) and regulatory T (Treg) cells may contribute to the pathogenesis of nasal polyps.

This study assessed the frequency of Th17 and Treg cells and related cytokines in patients with nasal polyps and tested for associations with mucosal remodeling.

Surgical samples from 12 controls and 33 CRSwNP patients were analyzed histopathologically. The frequency of Th17 and Treg cells in peripheral blood mononuclear cells (PBMCs) and tissues were determined using flow cytometry. Th17 and Treg cells-related cytokines in plasma were measured by Cytometric Bead Array (CBA) multiplex assays and enzyme-linked immunosorbent assays (ELISAs).

Eosinophilic CRSwNP (ECRSwNP) patients exhibited robust eosinophilia, whereas non-ECRSwNP patients were characterized by neutrophilia. Compared with non-ECRSwNP, an increased Th17/Treg ratio in ECRSwNP was associated with a less increased frequency of Th17 cells and a more striking reduction of Treg cells. An altered Th17/Treg cell ratio was positively correlated with eosinophilic and neutrophilic infiltration, submucosal basement membrane thickness, and the degree of subepithelial collagen deposition. Compared with non-ECRSwNP, ECRSwNP had higher levels of IL-17A and IL-4, and lower levels of IL-10 and TGF-β1, whereas non-ECRSwNP showed higher levels of IFN-γ and IL-6.

Th17/Treg cell imbalance in nasal polyps (both in tissues and PBMCs) with distinct cytokine profile may contribute to different inflammatory patterns (eosinophilic versus neutrophilic inflammation) and corresponding features of mucosal remodeling. Effective strategies can be designed to target a Th17/Treg imbalance to restore immune homeostasis in nasal polyps.

## INTRODUCTION

Chronic rhinosinusitis (CRS) is defined by persistent inflammation of the nasal and paranasal cavity mucosa that lasts ≥3 months. CRS with nasal polyps (CRSwNP), a multifactorial and highly heterogeneous disease, is clinically characterized by the appearance of polyps in the nasal cavity. CRSwNP causes typical symptoms, such as nasal obstruction, nasal discharge, and/or loss of smell.^1^ Despite aggressive medical therapy or endoscopic sinus surgical treatment, many patients tend to be poorly controlled and have a high reversion rate.^2,3^ Generally, CRS that is difficult to control is termed refractory chronic rhinosinusitis (RCRS). Several studies have focused on the histopathological and clinical characteristics of RCRS, which showed that different types of mucosal remodeling features, inflammatory patterns, and atopic conditions are neither involved in nor contribute to the pathogenesis of RCRS.^4,5^ Several factors that are correlated with a worse outcome or risk of recurrence of nasal polyps have been identified, such as high eosinophil infiltration (or high eosinophil density) and more severe preoperative disease (i.e., a higher CT score).^6^ For better management, CRSwNP can be classified as eosinophilic CRSwNP (ECRSwNP) or noneosinophilic CRSWNP (non-ECRSwNP) based on the dominant inflammatory cell type in tissues. In China, at least half of CRSwNP patients present as non-ECRSwNP.^7^ Non-ECRSwNP can be divided into several phenotypes and neutrophilia is considered to be the best-characterized phenotype in non-ECRSwNP (∼20–46% of non-ECRSwNP in Asia). Furthermore, increased neutrophilia in nasal polyps has been established to reduce responses to oral corticosteroid therapy, which makes these phenotypes of CRSwNP intriguing.^8^ As different inflammatory cell infiltration exerts different clinical effects on the treatment outcomes for nasal polyps, immune mechanism of inflammation of nasal polyps has become a hot research spot.

Th17 and Treg cell subsets, differentiated from naive CD4^+^ T-cells, play unique roles in controlling inflammatory response, with effector T-cell subsets driving immunity and inflammation and inhibitory Treg cells playing an opposing role by suppressing effector T-cells to limit excessive inflammatory responses.^9^ The balance of immunosuppressive Foxp3^+^ Treg cells and proinflammatory IL-17A^+^ Th17 cells represents a critical factor in the regulation of immune homeostasis. A reduction in Treg cells that remove a suppressive influence on Th2 function can directly lead to a Th2 cell-dominated immune reaction, which can result in increased eosinophilic tissue infiltration.^10^ Notably, inflammation in ECRSwNP is mainly regulated by a Th2 inflammatory milieu characterized by IL-4, IL-5, and IL-13, which are primary from Th2 cells.^11^ Several studies have detected impaired Treg cell functionality or an excessively low frequency of Treg cells in nasal polyps compared with control tissues.^7,12,13^ But these studies do not divide nasal polyps into different inflammatory patterns. IL-17A—an inflammatory factor with diverse functions that is mainly produced by Th17 cells—plays a major role in inflammation, cancer, host defense, and autoimmune disease.^14^ Notably, IL-17A can directly or indirectly induce the recruitment of neutrophils to sites of inflammation and positive correlations exist between the expression levels of IL-17A with neutrophil counts in airways.^15^ In Caucasian CRSwNP patients without cystic fibrosis, Derycke et al found that IL-17A could modulate neutrophil survival, highlighting the role of Th17 cells in neutrophilic inflammation in CRSwNP.^16^ Based on the previous findings, we speculate that different inflammatory patterns of nasal polyps are dominated by 1 subset or controlled by the imbalance of Th17/Treg cells.

Although current evidence supports the potential role of Th17 and Treg cells in determining the inflammatory state of CRSwNP, these studies just focus on 1 subset (Th17 or Treg cells) in one kind materials (tissues or blood).^7,12,13,16^ Besides, it still remains unclear to what extent this potential association between inflammation secondary to imbalanced of Th17 and Treg cells and mucosal remodeling occurs in ECRSwNP and non-ECRSwNP. Importantly, the balance of these 2 subsets of Th cells, which carry out opposing functions and exert reciprocal effects in the differentiation,^17^ has not fully evaluated in different phenotype of nasal polyps. Given the above considerations, this study was designed to explore the level of Th17 and Treg cells both in tissues and blood among patients with nasal polyps of different inflammatory patterns. We also examined the correlation between these 2 subsets and mucosal remodeling-related parameters. Th1/Th2/Th17/Treg cells related cytokins in plasma were also detected.

## MATERIALS AND METHODS

### Patients

Nasal mucosal tissues were obtained from 33 patients (19 men and 14 women) who were diagnosed with NP and were admitted for functional endoscopic sinus surgery at the Department of Otorinholaryngology, Head and Neck Surgery of Beijing Chaoyang Hospital at the Capital Medical University (Beijing, China) between January and July 2015. Diagnoses were made according to the criteria of the European Position Paper on Rhinosinusitis and Nasal Polyps 2007 (EP3OS 2007). CRSwNP was classified as eosinophilic when the frequency of tissue eosinophils exceeded 10% of total infiltrating cells/HpF.^7^ The control group included 12 patients undergoing septoplasty for anatomic variations, and uncinate tissues were removed during septal surgery and processed to tested as control tissues. All the patients were evaluated by a skin prick test using a standard panel of aeroallergens for the atopic status. The patients who were diagnosed with antrochoanal polyps, cystic fibrosis, fungal sinusitis, primary ciliary dyskinesia, or gastroesophageal reflux disease were excluded. The patients should stop use oral glucocorticoid and intranasal steroid sprays at least 3 and 1 months before surgery, respectively. All control group patients were nonsmokers without infectious nasal or sinus diseases and the patients who were diagnosed with nasal polyps were nonsmokers.

All patients provided written consent before surgery to allow for the use of biological specimens in biomedical research. All procurement experiments were approved by the Ethics Committee of Beijing Chaoyang Hospital, Capital Medical University (No. 15-department -17).

### Sample Preparation

A total of 10 mL heparinized peripheral venous blood was collected from each participant. Plasma was isolated from peripheral blood, divided into aliquots, and stored at –80°C until later use to measure cytokine concentrations. PBMCs were isolated by Ficoll density gradient centrifugation from peripheral blood within 2 hours. Tissue samples were divided into 2 sections; 1 section was fixed overnight in a freshly prepared fixative containing 4% paraformaldehyde in phosphate buffered saline (pH 7.4) and embedded in paraffin wax for hematoxylin and eosin (H&E), Masson's trichrome and immunohistochemistry staining; the other section was processed to obtain a single-cell suspension for flow cytometry analysis. Briefly, tissue samples were cut into small pieces and digested in RPMI-1640 medium with endotoxin-free collagenase I (2 mg/mL, Sigma–Aldrich, St Louis, MO) for 1 hour at 37°C. Digested tissues were filtered through a 100-μm cell nylon mesh (BD Bioscience, San Diego, CA).

### Histological Staining

Surgically procured tissue specimens obtained from the NP and control groups were formalin-fixed and paraffin-embedded. Paraffin sections that were 4-μm thick were stained with H&E, Masson's trichrome according to the manufacturer's instructions. Sections stained with H&E were used to determine the number of eosinophils, the basement membrane (BM) thickness, and degree of edema. Sections stained with Masson's trichrome were used to determine the thickness and deposition degree of subepithelial collagen. All parameters were assessed using an image analyzer (SP 500; Olympus, Tokyo, Japan). A total of 5 randomly selected FOVs were evaluated for each stain at a magnification of 400 × by 2 experienced pathologists.

### Immunohistochemistry

Paraffin sections of NP tissues and uncinate tissues were rehydrated and boiled in EDTA buffer (pH 8.0) for 10 minutes to retrieve antigen. To inhibit endogenous peroxidase activity, 3% H_2_O_2_ was used. Normal serum was used to block nonspecific binding of secondary antibody. After washing, tissue sections were incubated with rabbit antihuman myeloperoxidase (MPO) antibody (polyclonal, 1:100 dilution, Zhongshan Golden Bridge Biotechnology, Beijing, China) at 4°C overnight. MPO was detected using the polyglucosan-horseradish peroxidase complex method (Changdao Biotechnology, Shanghai, China) according to the manufacturer's instructions. Slides were developed using 39,39-diaminobenzidine, which rendered positively stained cells brown. Finally, sections were counterstained with hematoxylin and mounted. Sections treated with species-matched and isotype-matched antibodies served as negative controls. All the stained sections were examined by 2 independent observers who were blinded to the experimental and clinical date. Five fields were randomly selected and the number of positive cells per high-power field was analyzed.

### Flow Cytometry

Treg cells (defined as CD4^+^CD25^+^FoxP3^+^) were evaluated in unstimulated PBMCs and single-cell suspensions were stained for 30 minutes at 4°C with anti-CD4-PerCP-5.5 and anti-CD25-APC. For intranuclear staining, the Human FoxP3 Staining Kit (BD Biosciences) was used according to the manufacturer's instructions. Briefly, after fixation and permeabilization, cells were incubated for 30 minutes at 4°C with anti-FoxP3-PE. Th17 cells (defined as CD3^+^CD8^-^IL-17A^+^, the reason is that the downregulation of surface CD 4 molecules after stimulation with PMA and ionomycin) were identified by intracellular cytokine staining (ICS). Cells were stimulated in culture medium with PMA (50 ng/mL; Sigma–Aldrich) and ionomycin (1 mg/mL; Calbiochem) for 4 hours at 37°C; Golgi-stop (BD Biosciences) was also added to the restimulation media. Briefly, surface staining with anti-CD3-FITC and anti-CD8-APC was carried out for 30 minutes at 4°C. Then, ICS was performed according to the Cytofix/Cytoperm kit instructions (BD Biosciences), and cells were incubated for 30 minutes at 4°C with anti-IL-17A-PE. All monoclonal antibodies (anti-CD25, FoxP3, CD8, and IL-17A) and corresponding isotype controls were obtained from BD Biosciences. Stained cells were washed twice before analysis using a BD FACS Canto II flow cytometer (San Jose, CA).

Samples were immediately run on BD FACS Canto II flow cytometer and analyzed using BD FACS Diva software. Lymphocytes were selected in a plot of forward scatter (FSC) and side scatter (SSC). CD3^+^ and CD4^+^ events were gated in a CD3 versus CD4 dot plot. To identify Th17 cells, CD8 versus IL-17A dot plots were constructed. To evaluate Treg cells, CD25 versus FoxP3 dot plots were used. Results are expressed as the frequencies of CD3^+^CD8^–^IL-17A^+^/CD3^+^ and CD4^+^CD25^+^FoxP3^+^/CD4^+^ T-cells.

### Cytokine Detection

Levels of plasma cytokines (IFN-γ, IL-4, IL-17A, IL-6, and IL-10) were detected using a Human Th1/Th2/Th17 Cytometric Bead Array kit (BD Biosciences) according to manufacturer's instructions. Acquired data were analyzed using BD CBD calibration and analysis software. Values below the limit of detection on each plate were set to 0. Levels of TGF-β1 in plasma were measured by ELISA. A commercial Duo-Set ELISA kit was used to detect total concentrations of TGF-β1 (2.0–9000 pg/mL; R&D System, Minneapolis, MN) according to the manufacturer's instructions.

### Statistics

Statistical analyses were carried out using SPSS version 17.0 (SPSS Inc, Chicago, IL). Continuous variables were presented as means ± standard deviation, medians (with minimum–maximum or interquartile ranges), or n (%) according to data distribution characteristics. The Kruskal–Wallis *H* test was used to assess significant intergroup variability. The Mann–Whitney *U* 2-tailed test was used for between-group comparisons. Differences in frequencies between groups were examined using the χ^2^ test. Spearman's rank correlation was used to examine relationships between 2 continuous variables. *P* < 0.05 was used as a threshold for statistical significance throughout this study.

## RESULTS

### Characteristics of Enrolled Subjects

Overall patient demographic and clinical characteristics are summarized in Table 1. A total of 45 patients who met all of the specified inclusion criteria were enrolled in this study. No significant differences were identified for age, sex, the concurrent rate of atopy, or the absolute blood neutrophils count among controls and ECRSwNP and non-ECRSwNP patients. The absolute blood eosinophils count in the ECRSwNP group was significantly higher than that in the controls and non-ECRSwNP patients (*P* < 0.01 for each), but there was no significant difference between controls and non-ECRSwNP patients.

**TABLE 1:**
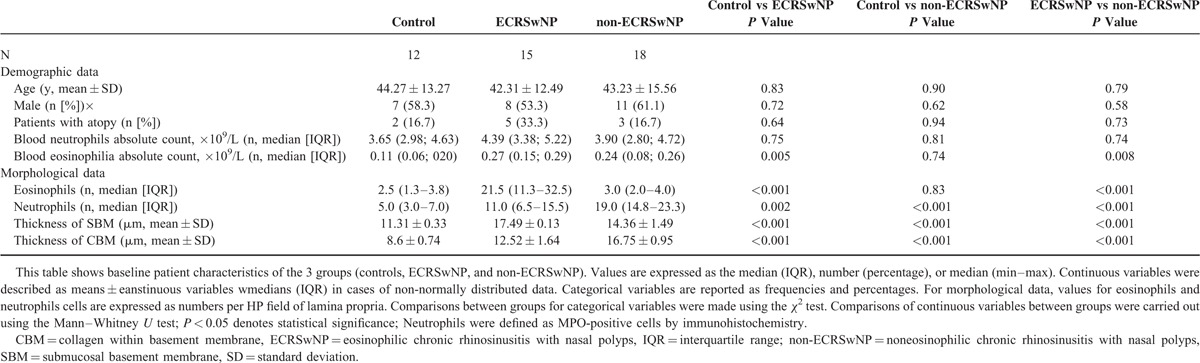
Baseline Characteristics of the Study Population

**FIGURE 1:**
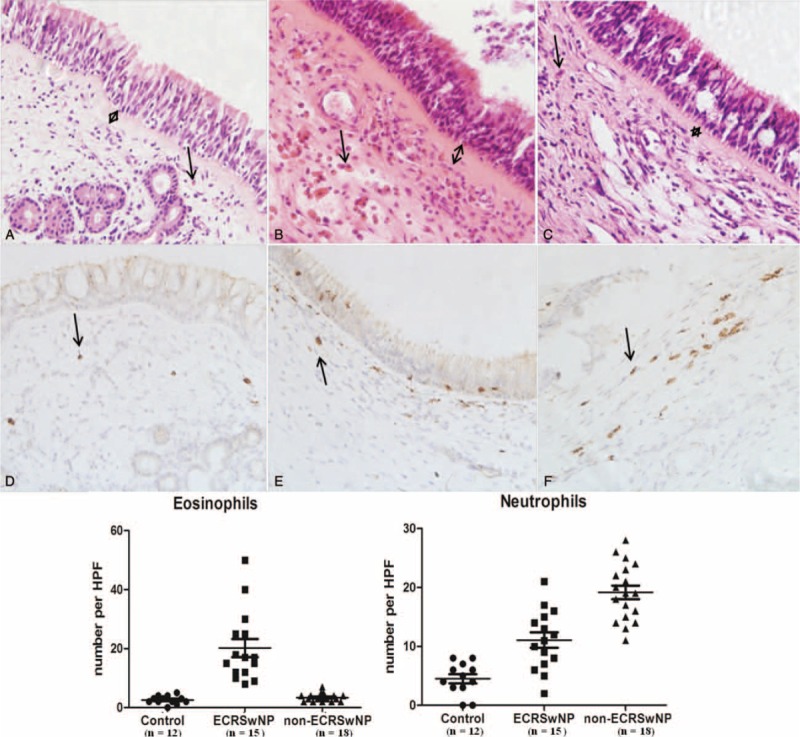
Histopathological sections of tissues from control subjects (A, D), ECRSwNP patients (B, E), and non-ECRSwNP (C, F) patients. H&E staining (A, B, and C show eosinophil indicated by arrows), immunohistochemistry (D, E, and F show MPO-positive cells indicated by arrows); original magnification, ×400. ECRSwNP = eosinophilic chronic rhinosinusitis with nasal polyps, H&E = hematoxylin and eosin.

**FIGURE 2:**
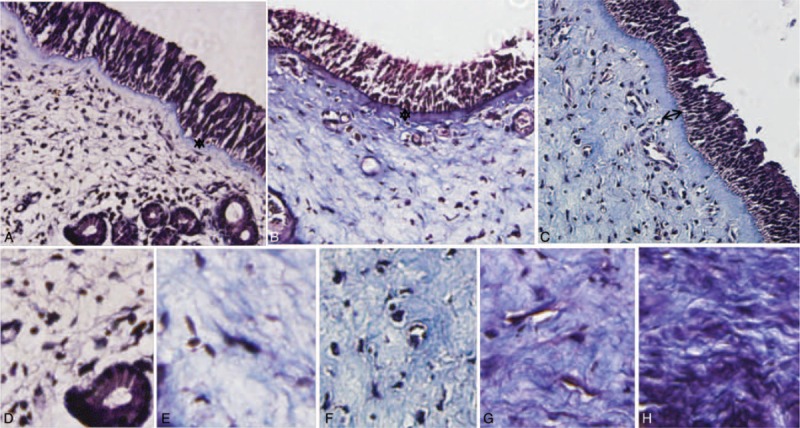
Histopathological sections from control subjects (A), ECRSwNP patients (B), and non-ECRSwNP (C) patients. Masson's trichrome staining (A, B, C), and typical examples of pathological grading scored from 0 to 4; panels D, E, F, G, and H show examples of 0, 1, 2, 3, and 4°, respectively; original magnification, ×400. ECRSwNP = eosinophilic chronic rhinosinusitis with nasal polyps.

Morphological data associated with remodeling parameters in control, ECRSwNP patients, and NECRSwNP patients are summarized in Tables 1 and 2 and Figures 1 and 2. Compared with controls and non-ECRSwNP patients, ECRSwNP patients exhibited a significantly higher frequency of eosinophils (*P* < 0.001); however, controls and non-ECRSwNP patients had similar frequencies of eosinophils. Compared with controls, neutrophils were significantly increased in both ECRSwNP (*P* < 0.01) and non-ECRSwNP (*P* < 0.001). Additionally, non-ECRSwNP presented with significantly higher levels of IL-17A compared with ECRSwNP (*P* < 0.001). In non-ECRSwNP patients compared with controls, the thickness of the SBM and SEC was markedly increased and there was a significant difference detected between all 3 groups (*P* < 0.001 for all). To compare differences in subepithelial collagen deposition among controls, ECRSwNP, and non-ECRSwNP, we graded the pathology of tissues sections on a scale of 0 to 4 for each group and calculated the mean rank of each group (Table 2). In non-ECRSwNP compared with controls, the mean rank was slightly, but significantly increased (*P* < 0.001). We also graded the edema degree on a scale of 0 to 2 for each group calculated the mean rank of each group (Table 2). Compared with controls, the mean rank of both ECRSwNP and non-ECRSwNP was significantly increased (*P* < 0.001) and there also existed significant difference between ECRSwNP and non-ECRSwNP (*P* < 0.001).

**TABLE 2:**
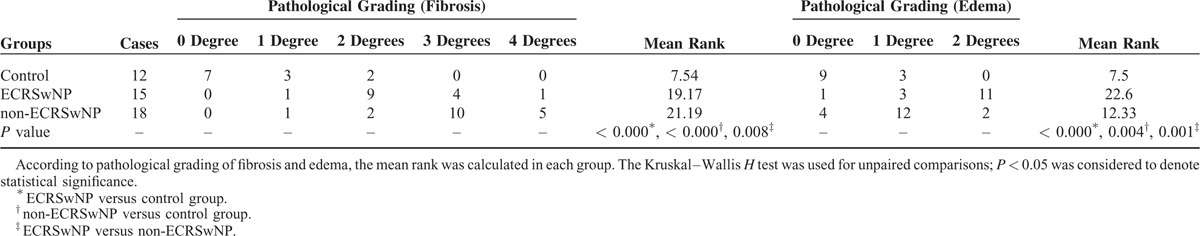
Subepithelial Collagen Deposition and Edema Scores of Sinus Tissues

### Altered Th17 and Treg cell Frequencies and Th17/Treg Ratios in Controls, ECRSwNP, and non-ECRSwNP

**FIGURE 3:**
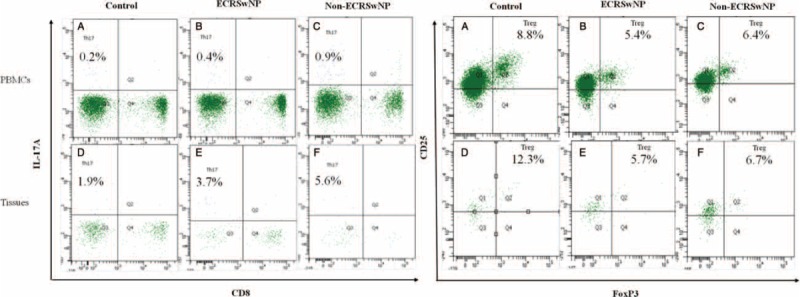
The ratio of Th17/Treg cells in PBMCs and tissues. The frequencies of Th17 cells (CD3^+^CD8^−^IL-17A^+^) and Treg cells (CD4^+^CD25^+^FoxP3^+^) from PBMCs and tissues were assessed by flow cytometry. PBMCs = peripheral blood mononuclear cells. ECRSwNP = eosinophilic chronic rhinosinusitis with nasal polyps.

**FIGURE 4:**
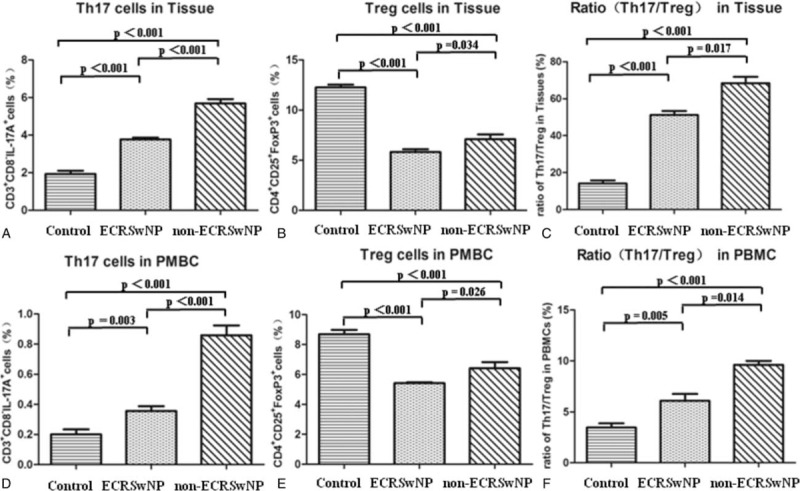
Th17 and Treg cell frequencies and Th17/Treg ratios in controls, ECRSwNP, and non-ECRSwNP. Data represent means ± SEM; *P* < 0.05 was considered to indicate statistical significance. ECRSwNP = eosinophilic chronic rhinosinusitis with nasal polyps, SEM = standard error of mean.

As shown in Figures 3 and 4, Th17 cells were defined as lymphocytes with a CD3^+^CD8^-^IL-17A^+^ phenotype. In non-ECRSwNP patients compared with controls, the proportion of Th17 cells in PBMCs and tissues gradually increased and there was a significant difference among the 3 groups (*P* < 0.01 for all). Treg cells are defined as CD4^+^CD25^+^Foxp3^+^ cells. Compared with controls, the frequency of Treg cells among PBMCs and in tissues was significantly reduced (*P* < 0.01 for all). Moreover, ECRSwNP patients exhibited a significantly lower frequency of Treg cells both in PBMCs and tissues compared with non-ECRSwNP patients (*P* < 0.05 for both). To investigate the association between Th17 and Treg cells, we analyzed the ratios of these cells both in PBMCs and tissues. In both PBMCs and tissues, the ratio of Th17/Treg cells between 3 groups existed significant difference.

### Associations Between Th17 and Treg cell Subsets and the Th17/Treg Ratio With Mucosal Remodeling-related Parameters

As shown in Table 3, Spearman's correlation analysis showed that the frequency of Th17 cells was positively correlated with the number of eosinophils (EOS; *r* = 0.425, *P* < 0.01) and neutrophils (NEU; *r* = 0.628, *P* < 0.01), the thickness of collagen within the basement membrane (CBM; *r* = 0.564, *P* < 0.05) and submucosal basement membrane (SBM; *r* = 0.534, *P* < 0.01), the degree of subepithelial collagen deposition (SCD; *r* = 0.572, *P* < 0.05), and edema (*r* = 0.401, *P* < 0.05). By contrast, the frequency of Treg cells was negatively correlated with the number of EOS (*r* = –0.626, *P* < 0.05) and NEU (*r* = –0.375, *P* < 0.05), the thickness of the SBM (*r* = –0.472, *P* < 0.05), and the degree of edema (*r* = –0.625, *P* < 0.01). Moreover, the ratio of Th17/Treg cells was positively correlated with the number of EOS (*r* = 0.481, *P* < 0.05) and NEU (*r* = 0.641, *P* < 0.01), the thickness of the SBM (*r* = 0.453, *P* < 0.01), and the degree of SCD (*r* = 0.427, *P* < 0.05).

**TABLE 3:**
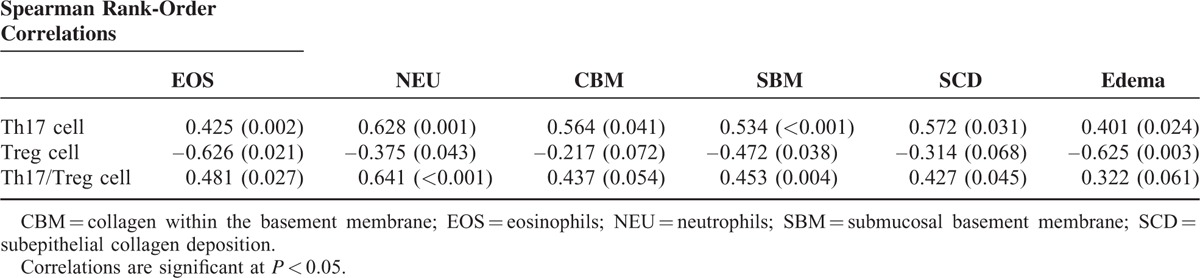
Correlations Between Th17 and Treg Cell Subsets and the Th17/Treg Ratio With Mucosal Remodeling-Related Parameters

### Th1/Th2/Th17/Treg Cell-Related Cytokine Secretion Profiles

**FIGURE 5:**
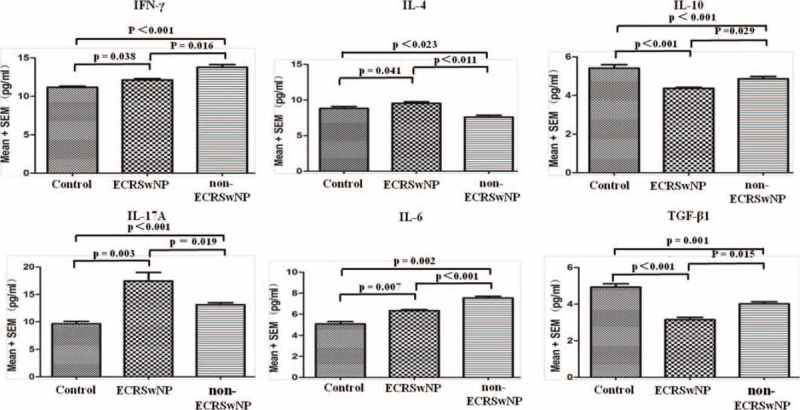
Th1/Th2/Th17/Treg cell-related inflammatory cytokines. Serum levels of IFN-γ (Th1), IL-4 (Th2), IL-17A (Th17), and IL-10 (Treg) are shown. Additionally, the serum levels of IL-6 and TGF-β1 (regulator of the Treg/Th17 balance) are shown. Data represent means ± SEM; *P* < 0.05 was considered to indicate statistical significance.

As shown in Figure 5, in non-ECRSwNP compared with controls, levels of IL-6 and IFN-γ significantly increased and there a significant difference existed among the 3 groups (*P* < 0.01 for all). Compared with controls, ECRSwNP patients presented with significantly higher levels of IL-4 (*P* < 0.05), whereas non-ECRSwNP patients showed significantly lower levels of IL-4 (*P* < 0.05); differences between ECRSwNP and non-ECRSwNP were significant (*P* < 0.05). Compared with controls, levels of IL-17A were significantly increased in both ECRSwNP (*P* < 0.01) and non-ECRSwNP (*P* < 0.01). Additionally, ECRSwNP presented with significantly higher levels of IL-17A compared with non-ECRSwNP (*P* < 0.05). Compared with controls, for both IL-10 and TGF-β1, both ECRSwNP and non-ECRSwNP patients exhibited markedly decreased cytokine levels (*P* < 0.01 for both). Moreover, ECRSwNP patients exhibited significantly lower levels of IL-10 and TGF-β1 compared with non-ECRSwNP patients (*P* < 0.05 for both).

## DISCUSSION

Herein, after directly comparing the morphological mucosal remodeling features of eosinophilic and noneosinophilic CRSwNP, we analyzed the frequency of Th17 and Treg cells both in tissue and PBMCs, calculated the ratios of these cells, and tested for correlations with parameters related to mucosal remodeling. The main finding of this study was that alterations in the frequency and ratio of the Th17 and Treg cell subsets could affect inflammatory patterns and features of mucosal remodeling in nasal polyps with a distinct cytokine profile.

Notably, in this study, CRSwNP was characterized by a markedly increased percentage of Th17 cells and a significantly reduced percentage of Treg cells both in PBMCs and tissues, which is mostly consistent with previous reports.^7,13,16^ We only found 1 report that directly compared the functionality of Th17 and Treg cell subsets in nasal polyps with different phenotypes.^7^ That study demonstrated that both CRSsNP and CRSwNP patients presented with impaired Treg cell function and enhanced Th1/Th2/Th17 effector responses within tissues. Moreover, ECRSwNP, but not non-ECRSwNP, was characterized by Th2-skewed inflammation with predominant Th17 cell polarization. However, in our present study, we found that changes in the Th17 and Treg cell subsets in ECRSwNP and non-ECRSwNP patients differed, resulting in a trend for an increased Th17/Treg ratio, which was likely associated with the inflammatory pattern of CRSwNP. The Th17/Treg ratio was significantly increased both in ECRSwNP and non-ECRSwNP patients. Moreover, the increased Th17/Treg ratio in ECRSwNP patients coincided with slightly increased (reduction in the increased) frequency of Th17 cells and a significantly decreased frequency of Treg cells. Additionally, the increased Th17/Treg ratio in non-ECRSwNP patients coincided with a significantly (more markedly) increased frequency of Th17 cells and a slightly (less) reduced frequency of Treg cells. Thus, these findings indicate a potential role for an imbalanced Th17/Treg ratio in the development of different inflammatory nasal polyps.

To further characterize the specific roles of Th17 and Treg cells in the pathogenesis of ECRSwNP and non-ECRSwNP, we assessed the effects of these CD4^+^ T-cell subsets on tissue EOS and NEU accumulation and related morphological features. The positive correlation coefficient (*r* = 0.628) between Th17 cells and neutrophilic infiltration in the subepithelia was greater than that between Th17 cells and eosinophilic infiltration in the subepithelia (*r* = 0.425), which suggested that Th17 cells contribute more strongly to neutrophilic than eosinophilic infiltration. By contrast, the inverse correlation coefficient between Treg cells and eosinophilic infiltration in the subepithelia (*r* = 0.626) was greater than that between Treg cells and neutrophilic infiltration in the subepithelia (*r* = 0.375), suggesting that an inadequate Treg cell response could contribute much more to eosinophilic than neutrophilic infiltration. Other previous studies have reported regulatory effects of Th17 and Treg cell functionality on the accumulation of EOS and NEU in nasal polyps.^16,18^ However, this report is the first to show that Th17 cells have higher association with neutrophilic infiltration than eosinophilic infiltration, whereas Treg cells show the opposite association. Notably, Th17 and Treg cells carry out opposing functions in the immune response, with Th17 cells driving immunity and inflammation and Treg cells limiting autoimmune and inflammatory responses.^9^ In other words, an inadequate Treg response and an excessive Th17 cell response would exacerbate inflammatory disease. Indeed, in nasal polyps, biased differentiation of CD4^+^ T-cells into Th17 and Treg cells resulted in an imbalance in Th17 and Treg cells, which lead to distinct inflammatory patterns (eosinophilic vs neutrophilic inflammation) in nasal polyps.

The relationship between inflammation and mucosal remodeling remains the subject of ongoing investigations. Herein, we directly analyzed the relationship between NEU and EOS numbers and remodeling parameters. After further analysis of ECRwNP and non-ECRSwNP patients, we found that ECRSwNP was dominated by eosinophilia, whereas non-ECRSwNP was dominated by neutrophilia. And we also found that 45.5% of CRSwNP patients exhibited eosinophilia in NPs, which was consistent in with previous studies conducted in East Asia (<50% of patients presented with noneosinophilic inflammation).^7,19^ Furthermore, we found that ECRSwNP was characterized by remarkable edema, a thickened SBM, and increased mast cell infiltration. By contrast, non-ECRSwNP was characterized by apparent subepithelial collagen deposition, a thickened CBM, and goblet cell hyperplasia. A previous study reported similar findings.^20^ Similarly, we found that Th17 cells were positively correlated with CBM, SBM, SCD, and edema, whereas Treg cells were inversely correlated with SBM and edema. One plausible hypothesis that may explain the association between Th17 and Treg cells and remodeling parameters is biased eosinophil and neutrophil/granulocyte accumulation in ECRSwNP and non-ECRSwNP patients, respectively, which was strongly associated with imbalanced Th17 and Treg cell subsets. Indeed, in our study, we found that an imbalanced ratio of Th17/Treg cells was positively correlated with eosinophilic infiltration, neutrophilic infiltration, SBM, and SCD, suggesting high association with mucosal remodeling. Previous evidence suggests that eosinophils and neutrophils play important roles in airway remodeling.^7,20^ In brief, eosinophilic inflammation contributed to more edematous lesions, whereas neutrophilic inflammation was more closely related to fibrotic lesions, consistent with our present findings.

Few studies have characterized the immune parameters of nasal polyps, or specifically the balance or imbalance of T-helper-cell (Th) subsets (Th1/Th2/Th17) and Treg cells.^7,13^ In our present study, we conducted a comprehensive cytokine secretion profile in plasma. In this study, non-ECRSwNP was dominated with a Th1 milieu (IFN-γ) and elevated IL-6 and TGF-β1 levels. By contrast, ECRSwNP presented with Th2 cytokine profile, insufficient Treg cells, and enhanced Th17 cell functionality. However, A study by Cao and colleagues^7^ showed no significant difference in IFN-γ and TGF-β1 expression in tissues between ECRSwNP and non-ECRSwNP, which is different from our results, but the expression of IL-17A was significant difference between 2 groups, which is consistent with our findings. The discrepancy between our results and those of Cao and colleagues may be due to the fact that our sample size of nasal polys (n = 33) was relatively small and regional disparity. Therefore, a further multicenter design study with large samples is needed to certify this. In this study, ECRSwNP and non-ECRSwNP present with a distinct cytokine profile.

Remodeling involves the process of specific, but potentially irreversible structural changes in tissues. Generally, although inflammation and remodeling are closely related, whether a direct causal relationship exists between these 2 processes remains unknown. In this present study, for the first time, we analyzed the frequency of Th17 and Treg cells both in tissues and PBMCs and related cytokines between ECRSwNP and non-ECRSwNP patients. Inflammation that is secondary to these immune disorders are associated with remodeling parameters, but further studies regarding the mediators that link T-cell phenotypes or ratios to pathological findings will be essential to better understand the underlying inflammatory mechanisms and remodeling processes.

Our study has several limitations. First, the sample size was relative small, large sample experiments were needed. Second, the percentage of Th17 and Treg cells analyzed in flow cytometry was too few. The actual function of these 2 subsets in different inflammatory patterns of nasal polyps needed further study. Third, this study did not explore the checkpoints for the differentiation of Th17 and Treg cells, it would be important to evaluate the regulators that could rectify the imbalance of Th17/Treg in the further research. Finally, this study did not fully involve mechanism research and further studies are required to elaborate on the molecular mechanisms responsible for our findings.

In conclusion, our present study suggests for the first time that the Th17/Treg cell imbalance in nasal polyps both in tissues and PBMCs with a distinct cytokine profile may contribute to different inflammatory patterns and corresponding features of mucosal remodeling.
