# Altered Th17/Treg Ratio in Nasal Polyps With Distinct Cytokine Profile: Association With Patterns of Inflammation and Mucosal Remodeling: Erratum

**DOI:** 10.1097/01.md.0000482729.66173.0b

**Published:** 2016-04-18

In the article “Altered Th17/Treg Ratio in Nasal Polyps With Distinct Cytokine Profile: Association With Patterns of Inflammation and Mucosal Remodeling”,^1^ which appeared in Volume 95, Issue 10 of *Medicine*, several typos appeared. The article has since been corrected online.
